# Histological Patterns of Skin Lesions in Tuberous Sclerosis Complex: A Panorama

**DOI:** 10.3390/dermatopathology8030029

**Published:** 2021-07-04

**Authors:** Marine Cascarino, Stéphanie Leclerc-Mercier

**Affiliations:** 1 Department of Pathology, Paris Saint-Joseph Hospital Group, 75014 Paris, France; marine.cascarino@gmail.com; 2 Reference Center for Genodermatoses (MAGEC Center), Department of Pathology, Necker-Enfants Malades Hospital, Paris Centre University, 75015 Paris, France

**Keywords:** tuberous sclerosis complex, hypomelanotic lesions, confetti skin lesions, shagreen patch, angiofibroma, cutaneous hamartoma, folliculocystic and collagen hamartoma, forehead fibrous plaque

## Abstract

Tuberous Sclerosis Complex (TSC) is a multisystem genetic disease characterized by cutaneous and extracutaneous hamartomas. The diagnosis is based on the association of major and minor criteria, defined by a consensus conference updated in 2012. The clinical examination of the skin is crucial because seven diagnostic criteria are dermatological: four major (hypomelanotic macules, angiofibroma or fibrous cephalic plaques, ungual fibromas, shagreen patches) and three minor criteria (confetti skin lesions, dental enamel pits, intraoral fibromas). Skin biopsy is commonly performed to assert the diagnosis of TSC when the clinical aspect is atypical. Histopathology of TSC cutaneous lesions have been poorly reported until now. In this article, we review the histologic features described in the literature and share our experience of TSC skin biopsies in our pediatric hospital specialized in genetic disorders. Both hypomelanotic lesions and cutaneous hamartomas (angiofibroma/fibrous cephalic plaques, ungual fibromas, shagreen patches) are discussed, including the recent entity called folliculocystic and collagen hamartoma, with a special emphasis on helpful clues for TSC in such lesions.

## 1. Introduction

Tuberous sclerosis complex (TSC) is a rare autosomal dominant disease characterized by cutaneous and extracutaneous hamartomas (kidney, eyes, heart, brain, lungs). The prevalence is around 1/20,000 in the general population and the incidence about 1/6000 to 1/10,000 live births [1]. Skin manifestations are present in almost 100% of the patients affected by TSC [2].

TSC results from an inactivating mutation in *TSC1* or *TSC2*, two genes encoding tumor suppressor proteins: hamartin and tuberin, respectively. These proteins belong to the m-TOR pathway. The *TSC1/TSC2* mutations lead to increased protein synthesis and cell growth [3]. Two third of the cases are sporadic forms. Mutations are not identified by conventional genetic testing in 10 to 25% of TSC patients, possibly be due to mosaicism: *TSC1/TSC2* mutation would be present in only some organs and only some cells within those organs, then a “second-hit” mutation inactivates the remaining wild-type copy of *TSC1/TSC2* [3].

The diagnosis is therefore based on the association of major and minor criteria, as defined by a consensus conference updated in 2012 [1] (Table 1).

The dermatologist has a central role in the diagnosis of TSC because cutaneous manifestations account for 4 (hypomelanotic macules, angiofibromas or fibrous cephalic plaques, ungual fibromas, shagreen patches) of 11 major and 3 (confetti skin lesions, dental enamel pits, intraoral fibromas) of 6 minor diagnostic criteria.

An early diagnosis is crucial for a better management, and each lesion has a typical age of onset, but TSC diagnosis is sometimes challenging in pauci-symptomatic forms.

The cutaneous lesions of TSC can be classified into:

1. Hypomelanotic lesions:
  - hypomelanotic macules
  - confetti skin lesions
2. Connective tissue nevi (cutaneous hamartomas):
  - periungual fibromas (PF),
  - angiofibromas (AF),
  - shagreen patches (SP),
  - forehead fibrous plaques (FFP),
  - folliculocystic and collagen hamartomas (FCCH).

In this article, we will describe the histologic spectrum of TSC lesions reported in the literature, discuss the usefulness of skin biopsy in TSC and try to give clues for the diagnosis of TSC to avoid delayed diagnosis and allow an earlier management of the patient.

## 2. Hypomelanotic Lesions

**Figure 1:**
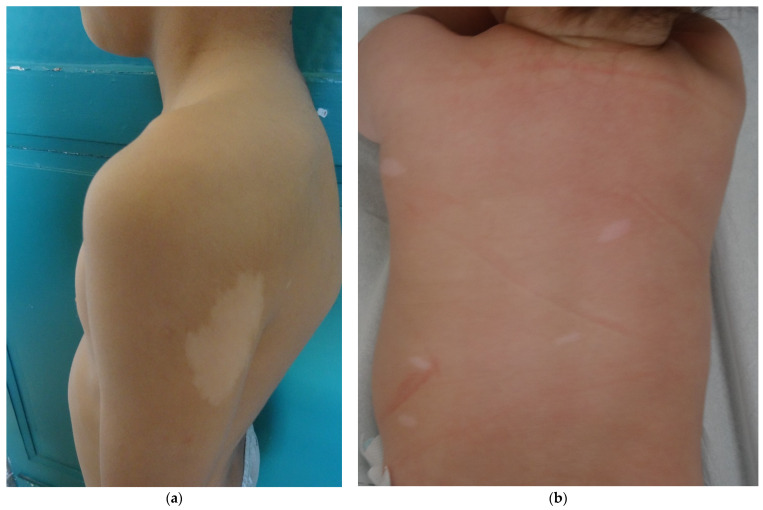
Hypomelanotic macules. (**a**): Ash leaf spot hypomelanotic lesion in a 7 year-old boy’s arm; (**b**): lance-ovate hypomelanotic macules in a 4 months boy’s back.

Hypomelanotic lesions are the most frequent and earliest lesions (90% of the patients, often seen at birth). The three most common aspects are polygonal, lance-ovate (or ash leaf spot) and confetti (guttate) (Figure 1a,b). Woods lamp examination is helpful for detecting these macules in infants. Hypomelanotic macules are asymmetrically distributed over the entire skin surface, and most commonly on the trunk and buttock [4]. Confetti macules are usually numerous, well circumscribed, small (1 to 3 mm in diameter) and diffuse, particularly seen over the forearms and lower legs. The presence of confetti lesions must alert the physician about a possible diagnosis of TSC.

Histopathologic studies showed a normal density of active melanocytes contrasting with a significant decrease in the amount of melanin pigment in the epidermis [4,5].

Electron microscopy demonstrates a reduced number of smaller, immature, and less melanized melanosomes, both in melanocytes and keratinocytes, due to the target of rapamycin complex (mTOR) pathway alteration, which is normally implicated in melanogenesis.

Recently, hypopigmented macules have been evaluated after topical rapamycin application (an inhibitor of mTor). Topical rapamycin led to a substantial improvement of hypopigmented macules and normalization of melanosome abnormalities in the treated skin [6].

## 3. TSC-Associated Cutaneous Hamartomas (Connective Tissue Nevi)

This item encompasses periungueal fibromas (PF), angiofibromas (AF), shagreen patches (SP), forehead fibrous plaques (FFP) and folliculocystic and collagen hamartomas (FCCH).

### 3.1. Angiofibromas (AF)

Angiofibromas are skin-colored to red-brown papules, typically on the central face. They usually appear at 3-years-old and their prevalence increases with age (8% in children younger than 2-years-old, increasing to 75% in children of 9-years-old and older) [7,8] The lesions progress in number with age and multiple AF appearing in childhood are characteristic of TSC [1,8]. The lesions progress also in appearance with age: early lesions are vascular macules and with time the fibrous component becomes more prominent, with dome-shaped smooth papules.

At least three AF must be present to be considered to be a major criteria, because one or two isolated lesions can be a normal finding [1].

AF histopathology has been described in three main articles [9,10,11]. AF share histopathologic similarities with fibrous papules of the face [12]. It presents as a dome-shaped lesion with a normal or sometimes hyperplastic epidermis. In the dermis, there is an expanding hypertrophy of the collagen tissue and the vascular structures. In older lesions, the collagen becomes denser and more sclerotic.

**Figure 2:**
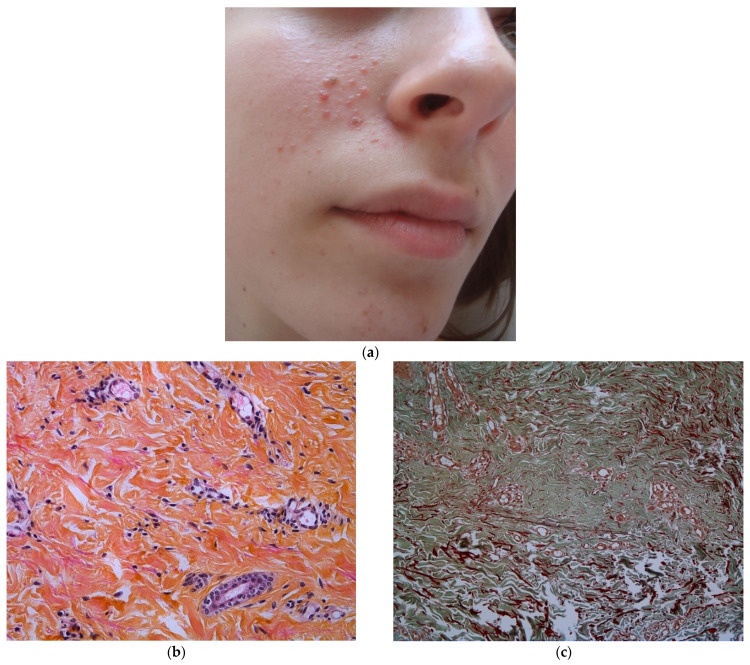
Angiofibroma. (**a**) Face’s angiofibromas in a 14 year-old patient; (**b**) Hematein eosin ×40: hypertrophy of the collagen bundles and of the vascular elements represented by dilated venules; (**c**). Orcein staining ×40: decreased and fragmented elastic fibers.

There is a tendency for a perifollicular arrangement of collagen, compressing the adnexal structures sometimes replacing them with an aspect of concentric collagen bundles. The vascular component is represented by widely dilated venules throughout the lesion. Staining for elastic fibers reveals a lack or decrease of elastic tissue in the AF, as in Figure 2b,c.

The cellular component consists of increased oval or stellate cells, tending to make clusters around the dilated blood vessels. Sometimes, multinucleated giant cells can be seen. This stellate cells are probably descending from dendritic cells, with Factor XIIIa antibody positivity [11,12].

Recently, NGS was performed on AF sample of TSC patient and demonstrated that a somatic second-hit mutation of the form CC > TT occurs in TSC facial angiofibroma at a high frequency, indicative of sunlight-induced DNA damage. The authors suggest that sun exposure could be responsible for a second hit event in *TSC1/TSC2* and therefore be responsible for the development of facial AF [13]. Photoprotection should indeed be strongly advised to TSC patients.

### 3.2. Periungual Fibroma (PF)

**Figure 3:**
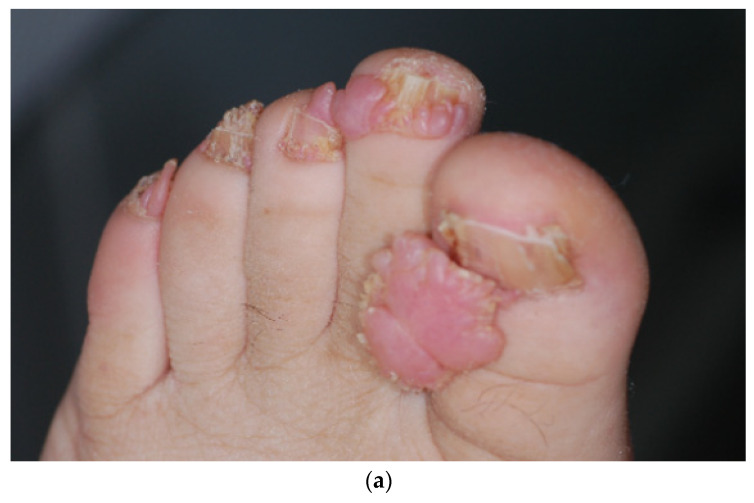

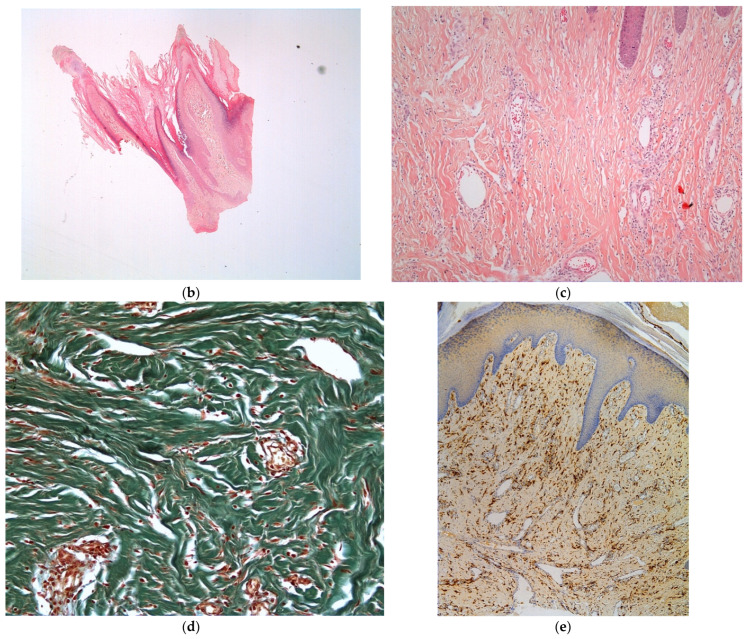
Periungual fibroma. (**a**). Clinical aspect of a toe periungual fibroma; (**b**). Hematein eosin ×5: exophytic dome-shaped lesion with hyperplastic epidermis; (**c**). Hematein eosin ×20: collagen bundles are vertically oriented; increased number of dilated venules; (**d**). Orcein staining × 40: decreased elastic fibers; (**e**). Immuno-histochemistry with anti-FXIIIa antibody showing positivity of stellate cells.

PF should be multiple (≥2) to be considered to be a TSC criteria, because post traumatic PF can also occur in the general population. They are located on the toes (90%) and/or fingers (56%) [14] (Figure 3a). Their frequency is 20 to 80% in older patients [1,7,8]. They are often painful and dysesthetic and then frequently removed.

In the literature, the histologic description is close to AF description and to idiopathic post traumatic PF [9,10]. The remarkable features distinguishing PF from AF are the vascular component and the arrangement of the collagen bundles. The vascular component is represented by an increased number of dilated venules lined by plumped endothelial cells. As in AF, the cellular component is made up of stellate cells with FXIIIa positivity (Figure 3e), usually not as numerous as in AF, and some multinucleate giant cells. Variable amounts of dense dermal collagen are present between the vascular spaces and collagen bundles, are vertically oriented and can even affect, in our experience, the hypodermis (Figure 3b,c). Elastic tissue is significantly decreased (Figure 3d).

### 3.3. Fibrous Cephalic Plaque (FCP)

**Figure 4:**
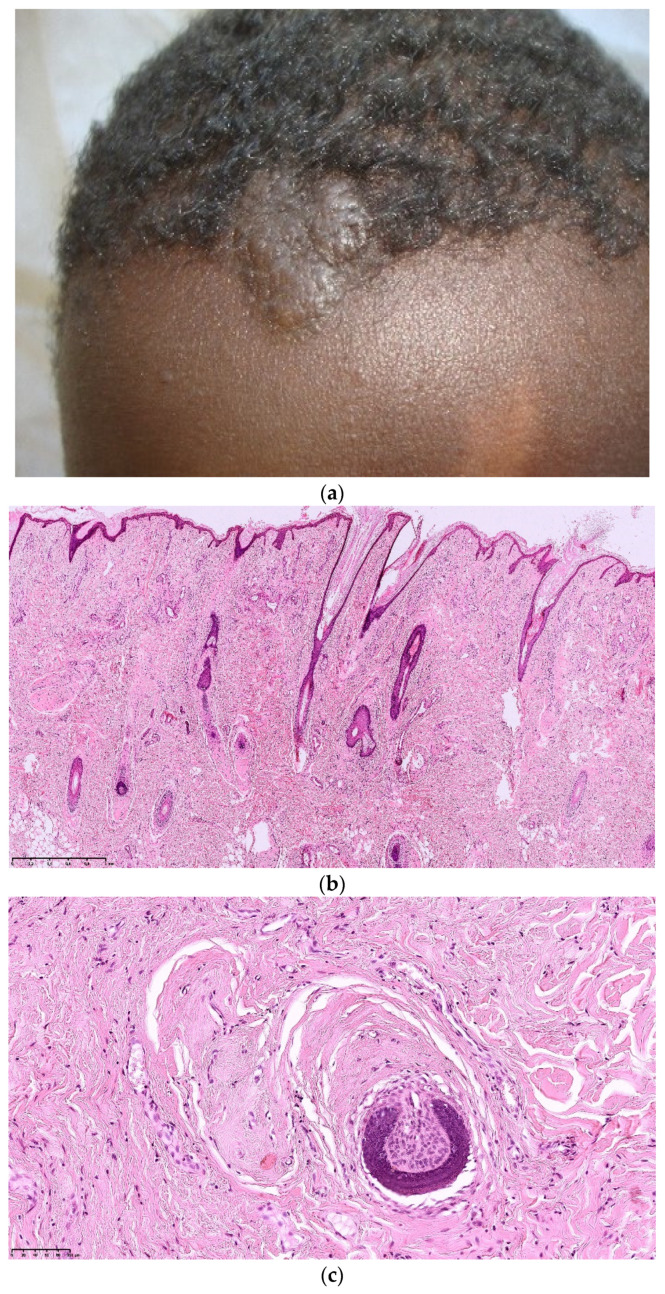
Fibrous cephalic plaque. (**a**): Clinical aspect of a fibrous cephalic plaque of the forehead; (**b**): Hematein eosin ×10: dense proliferation of collagen bundles in the dermis and the hypodermis; (**c**). Concentric perifollicular fibrosis leading to atrophy and compression of the follicle.

Fibrous cephalic plaques are observed in about 25% of TSC patients [1]. Formerly called forehead plaque, although often located unilaterally, they may be present on other parts of the face or scalp (Figure 4a). They can occur at any age. FCP are not frequently biopsied.

Pathological features of FCP described in the literature are similar to AF. The remarkable features distinguishing PF from AF are a more important prominent vascular dilatation and more sclerosis and hyalinization of the collagen with an aspect of concentric perifollicular fibrosis leading to atrophy and compression of the follicle [9] (Figure 4b,c). Recently, Traichel et al. described 13 FCP and noticed a FXIIIa positivity of the stromal cells and elastic fibers decreased [15].

### 3.4. Shagreen Patches (SP)

**Figure 5:**
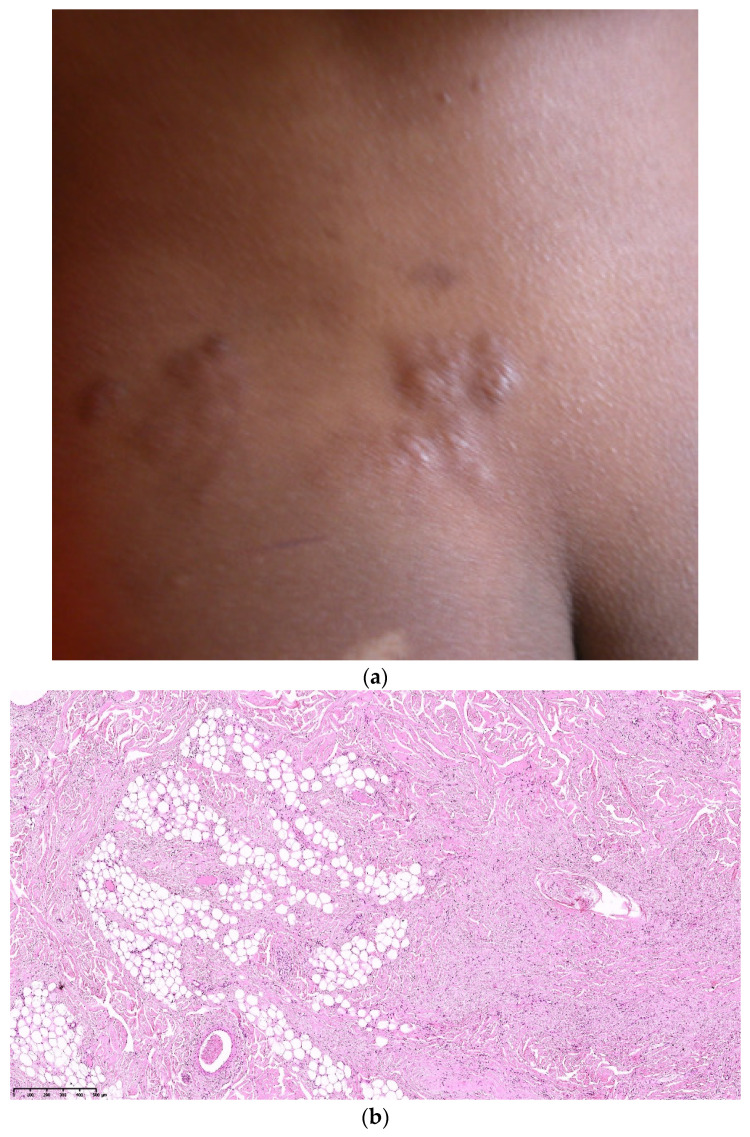

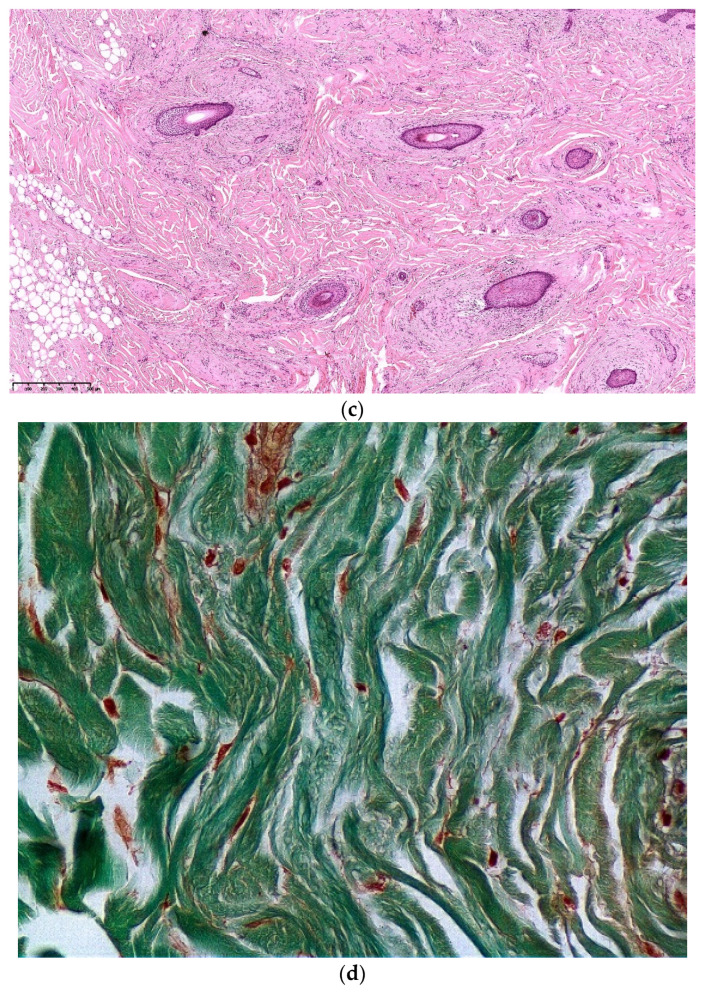
Shagreen patch. (**a**). Clinical aspect of a lumbar shagreen patch; (**b**). Hematein eosin ×20: extensive fibrosis with collagen bundles into the dermis and the hypodermis; (**c**). Hematein eosin ×20: concentric perifollicular fibrosis; (**d**). Orcein staining ×40: lack of elastic fibers.

Shagreen patches commonly present as large plaques on the lower back, with a bumpy or orange-peel surface, and this clinical aspect is almost specific of TSC [1,14]. (Figure 5a). They appear commonly in children in the first decade of life [7].

SP correspond histologically to collagenomas (or collagenic hamartomas). The dermis is replaced by a dense, relatively acellular and hyaline collagen which extends down to the subcutaneous fat (Figure 5b). As in AF and FCP, follicles are also involved with a concentric perifollicular collagen, atrophy and compression (Figure 5c). Some follicles are abnormal in shape. Sometimes, they are entirely replaced by a fibrous collagenic column. Elastic fibers are thin, fragmented or absent (Figure 5d) [9,16].

### 3.5. Folliculocystic and Collagen Hamartoma (FCCH)

FCCH is a new entity described in 2012 by Torrelo et al. with six cases in male patients with TSC [17]. Six additional cases of FCCH were reported [18,19,20,21,22,23], all occurring in patients with TSC, suggesting a causal relationship.

**Figure 6:**
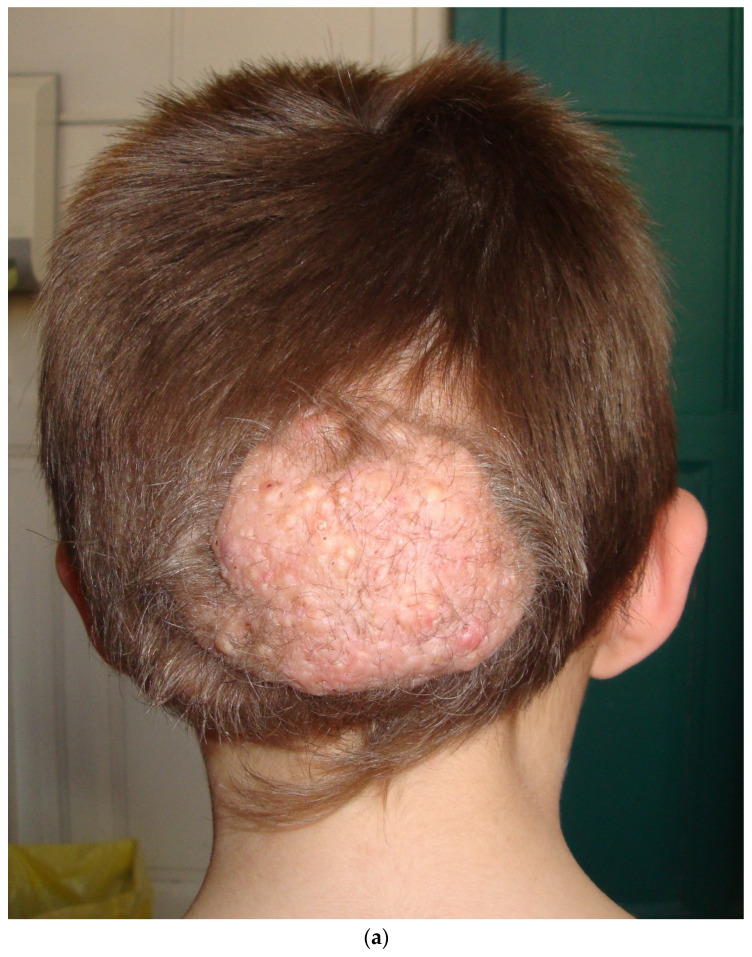

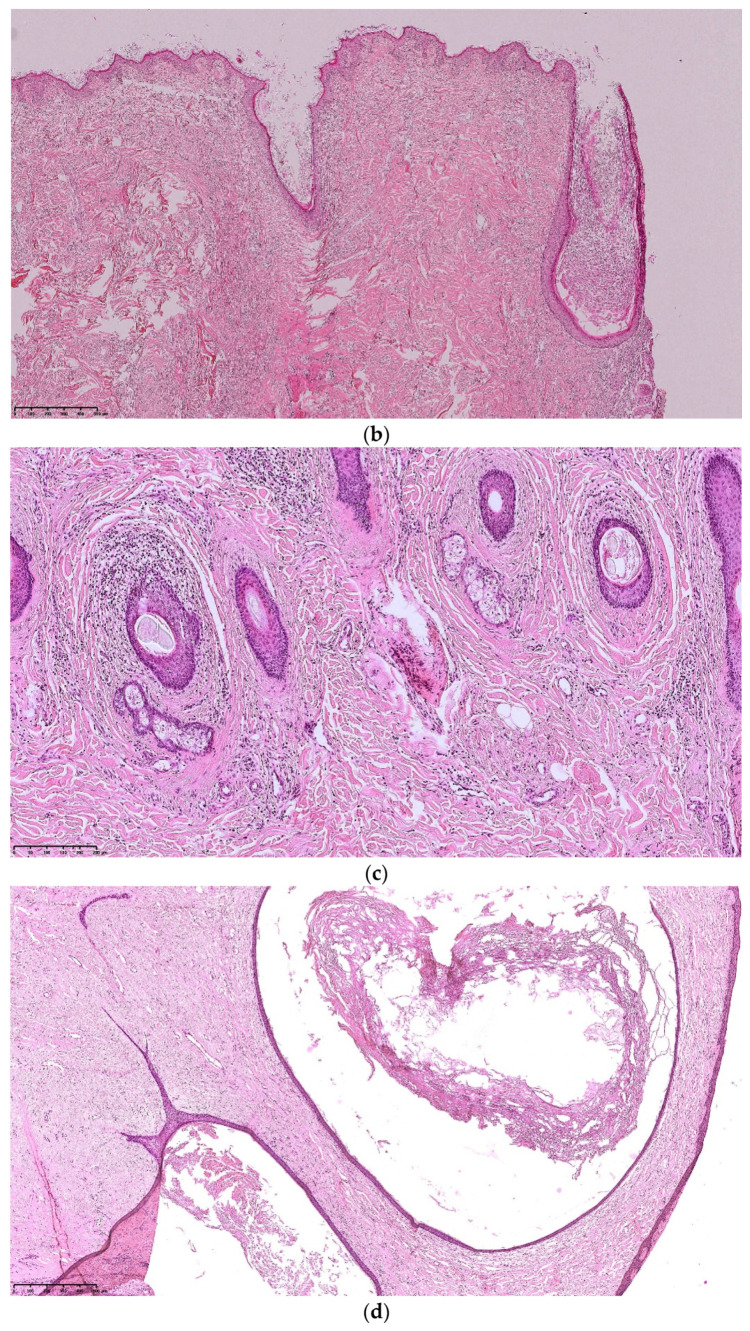
Folliculocystic Hamartoma. (**a**): clinical aspect of an occipital FCCH; (**b**): Hematein eosin ×10: extensive fibrosis and concentric perifollicular fibrosis; (**c**): Hematein eosin ×10: comedo openings; (**d**): Hematein eosin ×10: large infundibular cyst.

The clinical examination shows a solitary, painless, and large (several centimeters) infiltrated exophytic tumor, with an elastic consistence and an irregular surface covered by comedo-like structures (Figure 6a). This hamartoma is noticed at birth or during early infancy, mainly in boys, tending to occur on the scalp and the trunk.

Histopathologic examination of FCCH shows abundant and thickened collagen bundles occupying the whole dermis and extending into the subcutaneous fat (Figure 6b). There is also a marked concentric perifollicular fibrosis, surrounding hair follicles. This concentric fibrosis also involves the eccrine glands and surrounds some small and medium vessels.

A distinctive feature of FCCH is the presence of comedo-like formation and cysts lined by an infundibular epithelium, containing intact keratin (Figure 6c,d). Occasionally a ruptured cyst with foreign body reaction has been seen [17,21]. A vascular component has also been noticed in two cases with increased and dilated blood vessels [19,23]. Elastic fibers were not studied in the reported cases but in our experience, they are decreased or absent.

## 4. Other TSC-Associated Cutaneous Lesions

Café au lait spots are common, observed in 15 to 30% of CST patients, appearing in the first months of life. These lesions are not specific as they are seen in 16–19% of the general population [8,24,25].

Molluscum pendulum or acrochordons are found in 23% of patients with TSC [8]. However, these common lesions are also seen in the general population and have a non-specific histology (lesion with pedicle and fibrous axis without adnexa). Their necklace arrangement on the posterior neck could be a TSC sign [26].

Vascular cutaneous lesions, anemic nevus and Bier spots, are described in TSC patients in one study [27].

Recently, Lu et al. described the occurrence of juvenile xanthogranuloma in a 5 months old TSC patient [28]. Interestingly, Sirolimus (mTor inhibitor) had a significant effect on JXG and whole-exome sequencing in paraffin block tissue identified *TSC1* mutation.

## 5. Does TSC Cutaneous Hamartoma Belong to the Same Lesional Spectrum?

After the review of the literature and our retrospective examination of 20 lesions, we observed three common and constant components, more or less present in all varieties of TSC cutaneous hamartomas (AF, SP, FCP and FCCH):

- abundant thickened collagen, associated with adnexal involvement (concentric fibrosis)
- vascular hyperplasia,
- cellular proliferation of fibroblasts.

*TSC1* or *TSC2* mutations cause a defect in mTOR inhibition and promote cell proliferation but also angiogenesis and vessel modification due to increased production of VEGF by fibroblastic cells carrying the mutation [3,29].

We also noticed the association of decreased elastic fibers.

Among those three components, the concentric peri-follicular fibrosis with concomitant atrophy and compression of the skin adnexa seems highly suggestive of TSC.

This finding is not present in other types of collagenomas (sporadic or hereditary) in the literature and in our personal experience [30,31,32] (i.e., familial cutaneous collagenomas [33,34], Cowden syndrome [35,36], Proteus syndrome, Bushkle-Ollendorff syndrome [37,38], multiple endocrine neoplasia type 1 (NEM1) [39]). However, interesting way, this pattern is present in sporadic angiofibromas of the face [12].

In Birt-Hogg-Dubbé syndrome, skin lesions close to TSC are seen: fibrofolliculomas, trichodiscosomas and acrochordons [40]. They are generally multiple small skin-colored to grayish papules on the face. Pathological examination shows important follicular changes more pronounced than those observed on TSC cutaneous hamartomas [41,42]. There is no vascular hyperplasia and abundant or thickened collagen bundles are seen only around the involved follicle. In addition, TSC cutaneous hamartoma are much larger.

The observation of epidermal inclusion/cysts is unusual and seems to be a distinctive feature of FCCH [9,17]. Some authors suggested that the cysts are a component of the hamartoma, but we believe that the cystic aspect could be a consequence of the marked perifollicular fibrosis with a progressive dilatation of the upper part of the hair follicle.

Therefore, we believe that (like Cardona et al. [21] and Treichel et al. [15]), TSC-hamartomas such as FCP, AF, SP, and FCCH belong to a same histopathologic spectrum with the predominance of one or another component (Table 2).

## 6. In Conclusion, Is Histopathological Examination Useful in TSC Diagnosis?

Histopathology of TSC cutaneous lesions have been poorly reported until now and histology did not appear to be helpful for routine diagnosis.

**Figure 7:**
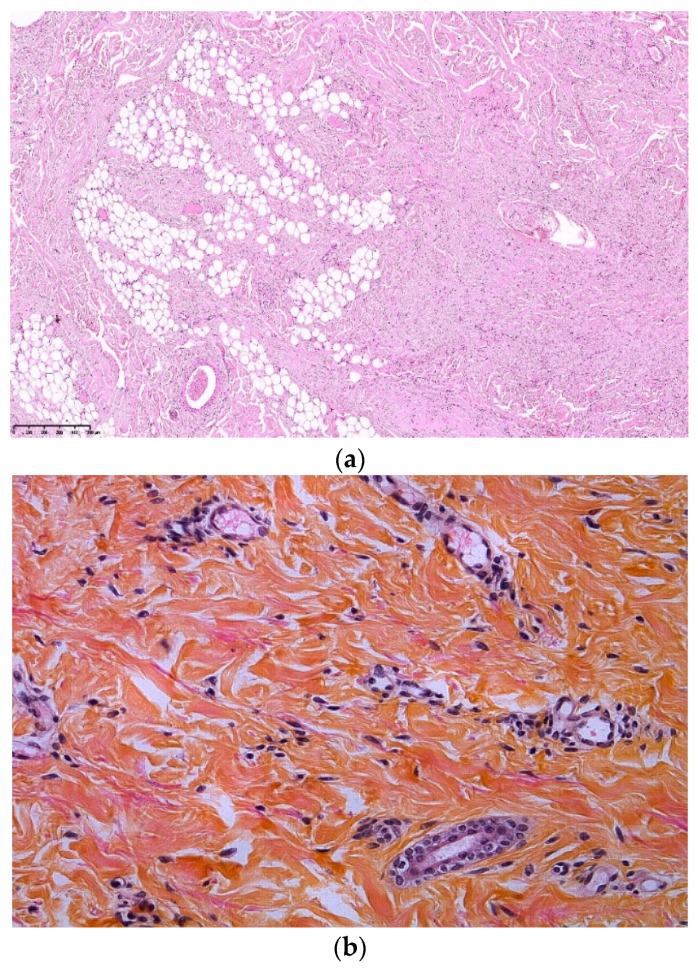

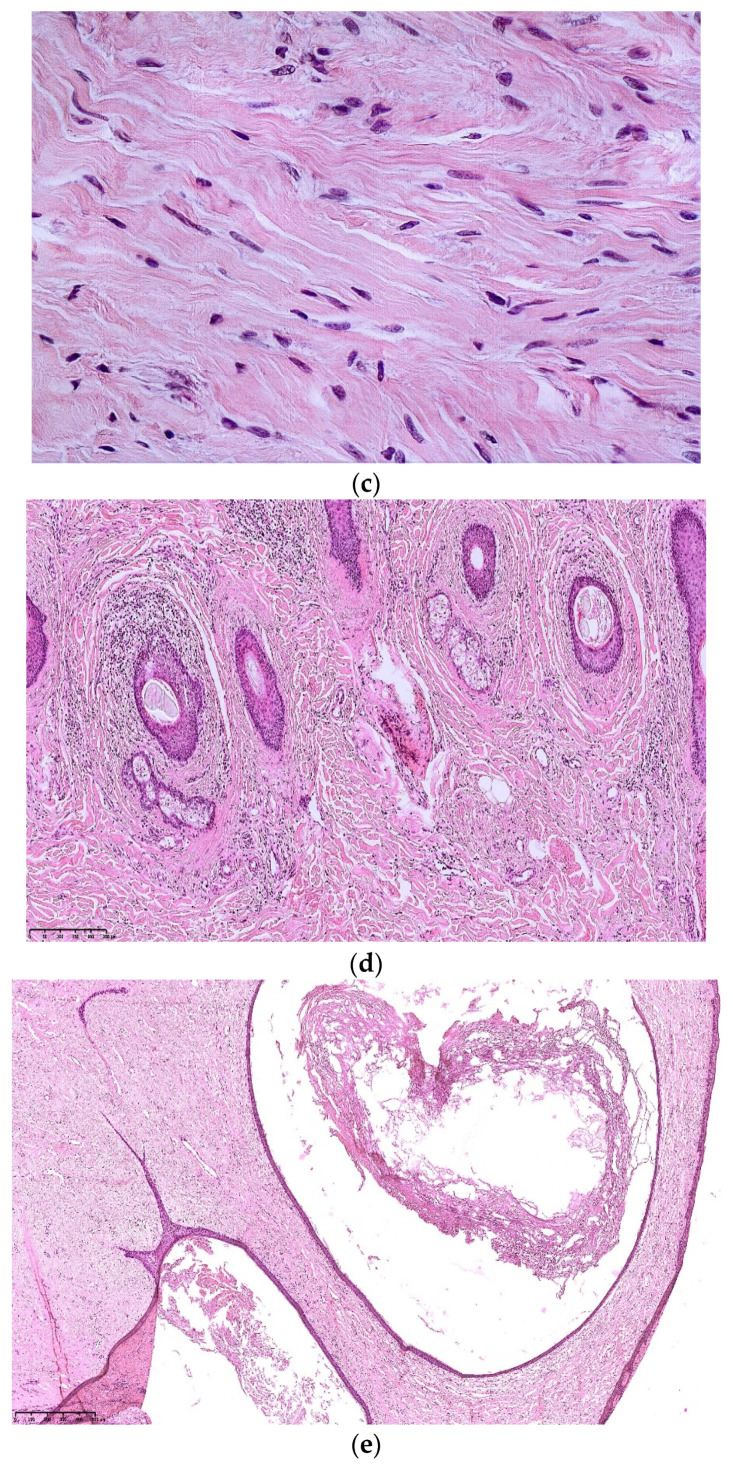

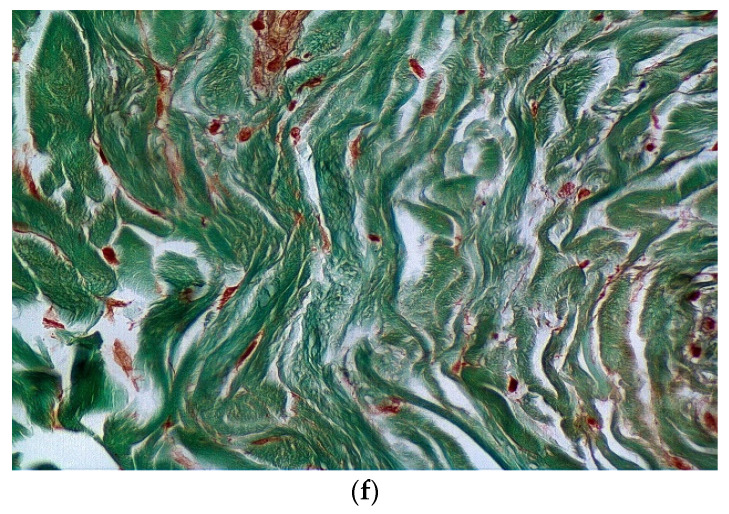
Common and distinctive histologic findings in TSC cutaneous hamartoma. (**a**): Hematein eosin ×10: fibrosis component: extensive fibrosis composed of thick collagen bundles; (**b**): Hematein eosin ×20: vascular component made of dilated vessels and thick collagen bundles; (**c**): Hematein eosin ×40: cellular component: stellate cells; (**d**): Hematein eosin ×10: Concentric perifollicular fibrosis; (**e**): Hematein eosin ×10: Dilated infundibular cyst; (**f**): Orcein staining: decreased or lack of elastic fibers.

Interestingly, all TSC-associated hamartomas such as AF, FCP, SP and FCCH belong to a continuous spectrum with common and distinctive histologic findings: abundant thickened collagen, sometimes with follicular involvement (perifollicular concentric fibrosis, and infundibular cysts for FCCH), vascular hyperplasia, fibroblast hypercellularity, and decreased elastic fibers (Figure 7).

FCCH seems to be specific entity of TSC, with a unique clinical aspect (irregular surface with comedo-like openings and cysts, a large size, occurring on the scalp or the trunk) and specific histologic findings (concentric fibrosis, infundibular cysts and comedo openings).

In pauci-symptomatic TSC, the histopathological features underlined above could be a strong argument for the diagnosis and allow the pathologist to suggest the diagnosis of TSC, conducting to an earlier diagnosis and a better management of the patient.

## Figures and Tables

**Table 1:** Diagnostic criteria for tuberous sclerosis complex 2012. Reprinted from ref. [1].

**A. Genetic diagnostic criteria**
Identification of either *TSC1* or *TSC2* pathogenic mutation is sufficient to make a definitive diagnosis of TSC
**B. Clinical diagnostic criteria**
**Major features:**
1. Hypomelanotic macules (≥3, at least 5-mm diameter)
2. Angiofibromas (≥3) or fibrous cephalic plaque
3. Ungueal fibromas (≥2)
4. Shagreen patch
5. Multiple retinal hamartomas
6. Cortical dysplasia
7. Subependymal nodules
8. Subependymal giant cell astrocytoma
9. Cardiac rhabdomyoma
10. Lymphangioleiomyomatosis
11. Angiomyolipomas (≥2)
**Minor features:**
1. «Confetti» skin lesions
2. Dental enamel pits (>3)
3. Intraoral fibromas (≥2)
4. Retinal achromic patch
5. Multiple renal cysts
6. Nonrenal hamartomas

Definite diagnosis: Two major features or one major feature with ≥2 minor features; Possible diagnosis: Either one major feature or ≥2 minor features.

**Table 2:** Histopathological pattern of TSC cutaneous hamartoma.

	Fibrosis	Cellularity	Dilated Vessels	Perifollicular Fibrosis	Decreased or Fragmented Elastic Fibers
Angiofibroma	+	++	++	0	++
	++ in old AF			+ in old AF	
Periungueal fibroma	+ to ++	+++	+++	0	++
Fibrous cephalic plaque	+++	+ to ++	+	++	+++
Shagreen patches	+++	+ to ++	+	+	+++
Folliculocystic and collagen hamartoma	+++	+	+	+++	+++

Note. +: low; ++: moderate; +++: intense.
